# Quackery in Paris

**Published:** 1859-01

**Authors:** 


QUACKERY IN PARIS.—There is now at Paris a negro who carries everything before him as a quack doctor.

He is a fine man of his race, covered with trinkets and diamonds, displaying great wealth in house, carriages, etc., and obtains the most fabulous fees from the easily-gulled Parisians. Among his various feats, *L'Union Médicale* relates the following:—He was sent for the other day into a very rich family, where a lady had for years suffered from very obstinate recurrences of fibrous tumors about various parts of the body. The best surgeons of Paris had failed in arresting the disease, and recourse was naturally had to wild systems of medicine; all these, however, including homœopathy, were powerless. Magnetism, necromancy, etc., had their turn, but nothing succeeded. At last, the negro's turn came. When he had cursorily examined the patient, he exclaimed, "This lady is curable, and I shall get her well in fourteen days." "Well, then," said the husband, "undertake the case at once." "My fee is £800; £240 is to be paid at once, and £40 on every other visit." Much demur was made to such a demand; but as the quack threatened to go away and leave the lady to her fate, he was allowed to pocket the £240, and has begun the treatment. The result is not known, but may easily be guessed at.—*The Lancet*, Oct. 2d, 1858.

Since the appearance of the above in the London *Lancet*, we learn that the impostor, after a short triumph, has disappeared from Paris, leaving many victims to mourn his loss.

